# Spatial sterol metabolism unveiled by stimulated Raman imaging

**DOI:** 10.3389/fchem.2023.1166313

**Published:** 2023-03-29

**Authors:** Yongqing Zhang, Yihui Zhou, Wen Fang, Hanlin Zhu, Cunqi Ye, Delong Zhang, Hyeon Jeong Lee

**Affiliations:** ^1^ Zhejiang Province Key Laboratory of Quantum Technology and Device, Department of Physics, Interdisciplinary Centre for Quantum Information, Zhejiang University, Hangzhou, China; ^2^ Key Laboratory for Biomedical Engineering of Ministry of Education, College of Biomedical Engineering and Instrument Science, Zhejiang University, Hangzhou, China; ^3^ Zhejiang Provincial Key Laboratory for Cancer Molecular Cell Biology, Life Sciences Institute, Zhejiang University, Hangzhou, China

**Keywords:** stimulated Raman scattering (SRS) microscopy, metabolite imaging, biophysics, cholesterol, genetic engineering, HMGCoA reductase, yeast

## Abstract

Spatiotemporal dynamics of small-molecule metabolites have gained increasing attention for their essential roles in deciphering the fundamental machinery of life. However, subcellular-level regulatory mechanisms remain less studied, particularly due to a lack of tools to track small-molecule metabolites. To address this challenge, we developed high-resolution stimulated Raman scattering (SRS) imaging of a genetically engineered model (GEM) to map metabolites in subcellular resolution. As a result, an unexpected regulatory mechanism of a critical metabolite, sterol, was discovered in yeast by amplifying the strength of vibrational imaging by genetic modulation. Specifically, isozymes of 3-hydroxy-3-methylglutaryl coenzyme A reductase (HMGR) were evident to promote ergosterol distribution to distinct subcellular locations, where ergosterol was enriched by a local HMGR-directed synthesis. The heterogeneity of this expression pattern thus provides new insights into sterol metabolism and related disease treatment strategies. These findings demonstrate SRS-GEM as a promising platform for new possibilities in investigating metabolic regulation, disease mechanisms, and biopharmaceutical research.

## Graphical Abstract

**Figure:**
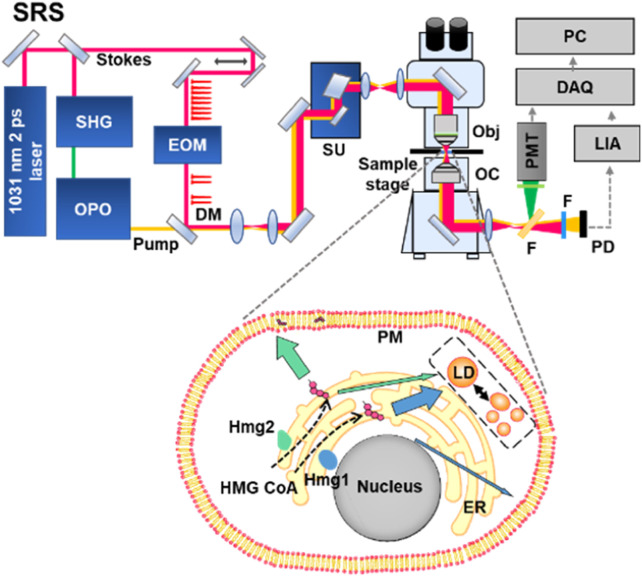
High-resolution stimulated Raman scattering (SRS) imaging of a genetically engineered model (GEM) enables metabolite imaging in a yeast model and uncovers an unexpected regulatory mechanism of sterol metabolism, providing new insights underpinning the distributional and functional importance of sterol in cells. SRS-GEM demonstrates a promising platform to explore unknown metabolic mechanisms beyond the reach of conventional approaches.

## Introduction

Maintaining metabolite homeostasis is critical for the function and survival of cells (Hotamisligil, 2006; Gillies et al., 2008). Metabolic regulations can happen at multiple levels, from single-cell, tissue, and inter-organ to systematic levels. In recent decades, significant progress has been made in understanding the regulatory mechanisms of metabolites such as lipids and glucose at tissue or inter-organ levels by mass spectrometry or nuclear magnetic resonance spectroscopy (Cameron et al., 1993; Dezaki et al., 2008; Peckett et al., 2011). Changes in regulations of metabolite transport and distribution under specific physiological conditions have also been studied systematically (Kishida et al., 2004; Yamauchi and Rogers, 2018; Yutuc et al., 2020), indicating that the function of metabolites is highly dependent on their spatial distribution. For example, distinct spatial localization of sterols was observed between brain regions (Yutuc et al., 2020). Others have elucidated the differences in metabolic and transport dysfunction of cholesterol and sterol intermediates between different tissues in patients with atherosclerosis and cancer (Yamauchi and Rogers, 2018). However, these studies mainly focus on the tissue or inter-organ level, and the spatial regulation of metabolites at the subcellular level remains relatively unclear due to the challenges of tracking small molecules *in situ* with such a high spatial resolution.

Cholesterol, a vital metabolite with multiple functions (Porter et al., 1996), was selected to demonstrate metabolite imaging. Dysregulation of cholesterol homeostasis significantly impacts various diseases (Kaul, 2003; Vance et al., 2005; Riscal et al., 2019). Cells can obtain cholesterol either by *de novo* synthesis or by uptake. Notably, the intestinal cholesterol pool size is influenced mainly by endogenous sources (800–1,000 mg) since the diet contributes only 300–400 mg of cholesterol per day (Mok et al., 1979), indicating the importance of endogenous cholesterol synthesis. The enzyme 3-hydroxy-3-methylglutaryl coenzyme A reductase (HMGR) catalyzes one of the most critical steps in cholesterol biosynthesis. Therefore, HMGR has become an important drug target in the treatment of hypercholesterolemia (Grundy et al., 2019), cardiovascular diseases (Zhou and Liao, 2009), and cancer (Jung et al., 2021). It thus triggered the development of a series of statins (e.g., lovastatin, atorvastatin, and simvastatin). However, the long-term use of statins has shown adverse effects (Ramkumar et al., 2016), which are linked to suppression of the mevalonate pathway and membrane damage (Sakamoto and Kimura, 2013), presenting an urgent need to identify a more specific way to control cholesterol homeostasis.

A reliable and controlled biological model is required to address this question, for which a yeast model was adopted. Yeast is a eukaryotic cell model widely used for the mechanistic study of cell metabolism, including cholesterol. Like cholesterol in mammalian cells, ergosterol, the counterpart of cholesterol in yeast, is synthesized *via* an HMGR-mediated pathway (Daum et al., 1998). It is mainly found on the plasma membrane and in lipid droplets (LDs) as ergosterol ester (Choudhary and Schneiter, 2012). However, unlike mammalian cells, yeast expresses two HMGR genes, namely, *HMG1* and *HMG2* (Basson et al., 1986). These two isozymes were found to regulate the proliferation of the endoplasmic reticulum (ER) membrane differently (Koning et al., 1996). Moreover, these two isozymes are degraded by different degradation pathways (Burg and Espenshade, 2011). These differences suggest that there may be a functional difference between these two isozymes.

Furthermore, although two HMGR isozymes guide an equal amount of carbon in the biosynthesis of sterols (Casey et al., 1992), it is not clear whether they have relatively independent regulatory mechanisms. Answering these questions would provide opportunities to develop approaches to regulate sterol metabolism more accurately. However, considering the small size of yeast (∼5 µm in diameter), spatiotemporal dynamics of sterol metabolism in yeast are less studied, primarily due to lacking practical tools for high-resolution sterol imaging. Thus, new metabolite imaging methods are needed to synergistically work with biological models to address novel and essential questions in life science and biomedical sciences.

Compared to conventional biochemical ensemble measurements, imaging approaches provide broad possibilities for tracking biomolecules. Molecular spectroscopy-based imaging methods started to gain increasing attention for the advantages of label-free selective imaging of small biomolecules, such as glucose, amino acids, fatty acids, and cholesterol, with high sensitivity and spatial resolution at the sub-micron scale. Recently, stimulated Raman scattering (SRS) microscopy has shown the capability of tracking small molecules based on their intrinsic molecular vibrational properties (Freudiger et al., 2008; Yue et al., 2014; Zhang et al., 2014; Cheng and Xie, 2015). A SRS signal pinpoints specific chemical bonds by excitation with its vibrational energy, providing *in situ* concentration information and allowing high-speed imaging of various biomolecules at the subcellular level (Min et al., 2011; Chung and Potma, 2013; Cheng and Xie, 2015; Karanja et al., 2017).

The new spectroscopic dimension came with a price—the acquisition speed. In principle, spectroscopic imaging should acquire at Nyquist frequency over the spectroscopy dimension and the XYZ of spatial dimension, providing a complete spectrum at each voxel. However, such a time-consuming acquisition would preclude studying living systems. Thus, methods to break the physical acquisition speed limit have been developed, including frequency spectral multiplexing (Fu et al., 2012; Liao et al., 2015), matrix completion using Lissajous scan (Lin et al., 2018), and compressive sensing (Berto et al., 2017; Lin et al., 2021). Alternatively, molecular tracking was realized by focusing on characteristic spectral bands, such as isotopic labeling (Freudiger et al., 2008; Zhang et al., 2011; Wei et al., 2015) and Raman tags (Wei et al., 2014; Lee et al., 2015; Wei et al., 2016; Du et al., 2022), that have unique spectral peaks in the spectrally silent region of natural compounds. However, the additional complexity of these methods precluded the broad impact of the technique.

**FIGURE 1:**
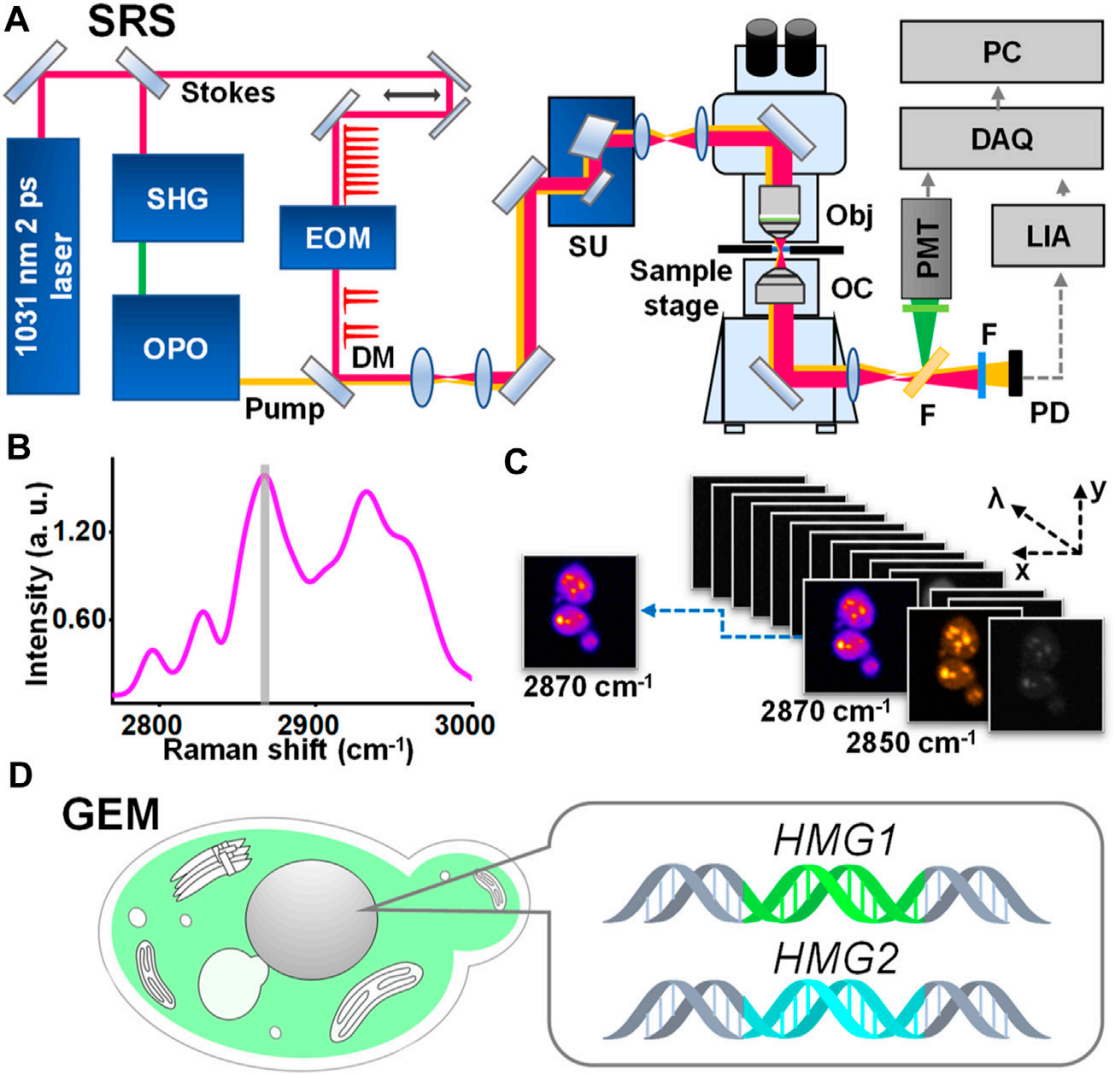
Schematic representation of SRS-GEM. **(A)** SRS platform. SHG, second harmonic generation; OPO, optical parametric oscillator; EOM, electro-optic modulator; DM, dichroic mirror; SU, scan unit; Obj, objective; OC, oil condenser; F, filter; PMT, photomultiplier; PD, photodiode; LIA, lock-in amplifier; DAQ, data acquisition; PC, personal computer. **(B)** Raman spectrum of ergosterol. The gray area highlights the characteristic peak selected for visualizing ergosterol. **(C)** Hyperspectral imaging of wildtype yeast. **(D)** Schematic representation of the GEM.

Here, we address the grand challenge by developing stimulated Raman imaging of a genetically engineered model (SRS-GEM) (Figures 1A–D). Although complicated, specific subcellular structures or locations are known to have a relatively stable composition in a model organism, allowing quantitative analysis based on the characteristic spectral peaks of the target molecule. Therefore, focusing on their distinctive bands with the proper excitation bandwidth would allow high-throughput tracking of specific metabolites. Demonstrated experimentally, high-selectivity imaging of subcellular metabolites reveals the functional differences of genes based on their spatial preferences.

Unexpectedly, the Hmg1 and Hmg2 genes in the *Saccharomyces cerevisiae* model showed different spatial preferences for sterol synthesis. Interestingly, there is a significant difference in the subcellular distributions of Hmg1 and Hmg2, consistently correlated with their functions. Furthermore, the subcellular regulation of sterol and lipid metabolism plays a decisive role in controlling cell growth and survival. The SRS-GEM platform collects insights into subcellular metabolism regulation for life science, synthetic biology, and disease treatment.

## Materials and methods

### Wildtype and HMGR knocked-out yeast strains

All the yeast strains used in this study are isogenic to the prototrophic CEN.PK strain of the budding yeast *S. cerevisiae* (van Dijken et al., 2000). Single-gene deletion mutants, *hmg1Δ* and *hmg2Δ,* were constructed by replacing each open reading frame with an HYG cassette using homologous recombination. The mutants are selected against hygromycin B and verified by the polymerase chain reaction (PCR) (Supplementary Figure S1). Cells were cultured in a synthetic defined (SD) medium containing 0.17% yeast nitrogen base without amino acids and ammonium sulfate (Difco), 0.5% ammonium sulfate (Sigma), and 2% glucose.

Wildtype, *hmg1Δ*, and *hmg2Δ* cells were cultured in SD medium overnight and diluted to 0.1 of A600 in fresh SD medium. Then, 100 μM simvastatin was added to logarithmically growing cells. Cells were harvested and fixed after 6 h of simvastatin treatment. Yeast cells were fixed with 1% formaldehyde at room temperature for 15 min and quenched with 125 mM glycine. The cells were pelleted by centrifugation at 3,000 *g* for 5 min at 4°C, washed twice with cold sterile water, and re-suspended in water for microscopic imaging.

### HMGR-GFP yeast strains

Yeast cells expressing Hmg1-GFP and Hmg2-GFP along with Mrh1-mCherry (a plasma membrane marker) or Nup157-mCherry (a nucleus marker) were cultured in synthetic glucose (SD) medium overnight and then diluted to 0.1 of A600 in fresh SD medium for another 6 h culture at 30°C. The localization of Hmg1-GFP and Hmg2-GFP in logarithmically growing cells was observed using a DeltaVision microscopy imaging system.

### Specimen preparation for imaging

Agarose gel was used for cell imaging. To prepare the gel, 10 µL of melted 3% (w/v) agarose solution (agarose solution prepared by dissolving agarose in distilled water) was pipetted on a glass slide and then immediately covered with a coverslip. After the gel is solidified, the coverslip was gently removed by rotating. Strips of a double-sided tape were mounted around the gel to help with the sealing after the sample was mounted on the gel. Cells were pelleted and washed with 1 × PBS twice. A volume of 2 µL of the cells was transferred to the agarose gel and covered with another coverslip. Gentle pressure was applied to ensure the sample was completely sealed.

### SRS microscopy

As for the microscope setup, we used an upright microscope (BX51WI, Olympus) coupled with a scanning galvanometer in *X* and *Y* directions (GVS002, Thorlabs) and a 60 × 1.2 NA water objective (Olympus, Japan). A picosecond laser (picoEmerald, Applied Physics & Electronics, Germany) supplied a synchronized pump beam (tunable wavelength 700–990 nm and 80 MHz repetition rate) and Stokes beam (fixed wavelength at 1,031 nm and 2-ps pulse width). The Stokes beam was modulated at 20 MHz using an electro-optic modulator. The forward-going pump and Stokes beams through the samples were collected using a high numerical aperture oil condenser (numerical aperture 1.4, Olympus). A short-pass dichroic mirror (650 nm cutoff, Thorlabs) was used to separate Raman photons and fluorescence. Fluorescence was acquired by using PMT (H7422-40, Hamamatsu, Japan) with a bandpass filter. A short-pass filter (1000 nm SP, Thorlabs, United States) was used to block the Stokes beam and transmit only the pump beam onto a large-area photodiode (10 mm × 10 mm) for the detection of the SRS signal. The detected photocurrent was sent into a lock-in amplifier. The extracted SRS and fluorescence signals were sent to the data acquisition unit. The final images were assembled in a home-built LabVIEW program during laser scanning at a rate of 10 μs per pixel.

### Spectral excitation bandwidth simulation

Peak fitting of ergosterol was handled by Origin 2018. Gaussian functions with a spectral resolution of 2 cm^-1^ to 80 cm^-1^ were, respectively, established by MATLAB to simulate the beams’ convolution.

### Image analysis

Images were processed and pseudo-colored by ImageJ. For SRS images, corresponding off-resonance images were taken at 2,100 cm^−1^ and subtracted from the on-resonance images, and the multi-spectral channels were assigned different colors. Quantification of metabolites was carried out by applying automatic local thresholding, analyzing particles, and profile measurements to the multi-spectral image stack. The results of the dual-color analysis were obtained from the intensity difference extracted by subtracting SRS intensity at 2,850 cm^−1^ in LDs and the plasma membrane based on SRS intensity at 2,870 cm^-1^ and then normalizing the intensity difference of different yeast treatments.

### Quantification of plasma membrane ergosterol levels from SRS images

Background plus three standard deviations of the background were taken as the starting point of the plasma membrane. The maximum signal in the membrane was taken as the midpoint of the membrane. Finally, the sum of the whole membrane signal was calculated, as shown in Supplementary Figure S2 in the highlighted area. For cells where the maximum plasma membrane signal is difficult to be determined, the cell membrane width was replaced by the average membrane width of other cells. Specifically, six points were selected equidistantly throughout the whole membrane for analysis (Supplementary Figure S2A), and 4–5 cells were analyzed in each group.

### Confocal Raman spectroscopy

The confocal Raman spectra of ergosterol, protein, and lipid were measured using ergosterol (powder), bovine serum albumin (powder), and olive oil as standard samples. Raman spectra were excited by a 785-nm laser source and obtained using the spectrometer (inVia Reflex, from Renishaw, Britain) with a spectral resolution of <1 cm^-1^.

### Determination of ergosterol levels by liquid chromatography–mass spectrometry (LC–MS)

Yeast cells were collected and disrupted in 100% ice-cold methanol using glass beads for lipid extraction. Total yeast lipids were extracted with chloroform/methanol (2:1) (v/v), as described previously (Ye et al., 2013), and dried using a CentriVap Concentrator system (Labconco, United States). Lipids were re-suspended in an MS-grade buffer (isopropanol: acetonitrile: water = 2:1:1) and cleared with two rounds of centrifugation before injection. Ergosterol levels were quantitatively analyzed in a positive ion mode by multiple reaction monitoring (MRM) acquisition using a triple quadrupole mass spectrometer (Triple Quad 6500+, AB SCIEX) that is coupled with high-performance liquid chromatography. Ergosterol was monitored with a transition of the precursor ion to the production (m/z 379.400→69.400) and separated chromatographically on a C18 column (ACQUITY UPLC BEH C18 Column, 130 Å, 1.7 μm, 2.1 mm × 50 mm). The flow rate was set to 0.15 mL/min using the following buffer system and LC method: Buffer A: 5 mM ammonium acetate in 33.3% methanol, 33.3% acetonitrile, and 33.3% water; Buffer B: 5 mM ammonium acetate in 100% isopropanol. T = 0 min, 0% B; T = 1 min, 20% B; T = 3 min, 60% B; T = 13 min, 98% B; T = 13.10 min, 20% B; and T = 16 min, 20% B. The retention time was compared to the ergosterol standard. The area under each peak was quantitated using SCIEX OS software and re-inspected for accuracy.

### Statistical analysis

For statistical analysis, a two-sample *t*-test was used to compare the metabolic difference between allelic types, including the ergosterol intensity, LD intensity, LD number, and cell area.

## Result

### SRS imaging enables label-free subcellular mapping of ergosterol

To tackle the challenges of metabolite imaging of the GEM, we developed a high-resolution, high-sensitivity SRS imaging platform (Figure 1A; Supplementary Figure S3). Briefly, a synchronously pumped optical parametric oscillator system provides picosecond pulse sequences at two different wavelengths for molecular vibrational excitation. These are fed into a home-built laser scanning microscope, followed by using a customized amplified detector and lock-in amplifier system. The technical advances include 1) a synchronized electro-optic modulator that locks to the laser repetition to ensure an accurate 2-pulse-on and 2-pulse-off modulation with 100% modulation depth; 2) a customized detector and lock-in amplifier with shot-noise limited detection, using the X channel (in-phase) signal to improve SNR by √2 of the R channel (amplitude), which also remove parasitic signals such as photothermal or cross-phase modulation; 3) a home-built microscope with a fixed objective lens to avoid a typical “dangling” arm, removing mechanical vibrations in the *Z*-direction critical for high-resolution imaging.

To demonstrate the chemical specificity of our system, a mathematical model was built to quantify and search for an optimal signal-to-background ratio (SBR, see Supplementary Figure S4). The Raman peak width of common small-molecule metabolites is about 25–40 cm^-1^, such as glucose (42 cm^-1^ at 1,373 cm^-1^) (Söderholm et al., 1999), ergosterol (38 cm^-1^ at 2,870 cm^-1^), and deuterated lipids (∼20 cm^-1^ at 2,103 cm^-1^) (Argov et al., 2008; Fu et al., 2013; Zhang et al., 2013; Zhang et al., 2013; Huang et al., 2020; Chen et al., 2021; Okotrub et al., 2022). The resulting SBR targeting ergosterol indicated high selectivity by <20 cm^−1^ narrowband excitations (Supplementary Figure S4D). However, a too narrow excitation band would result in a drastically low signal due to the multiphoton process of SRS. Thus, our system, with an excitation bandwidth of 14 cm^-1^, stands at an optimal balance between the signal level and selectivity.

**FIGURE 2:**
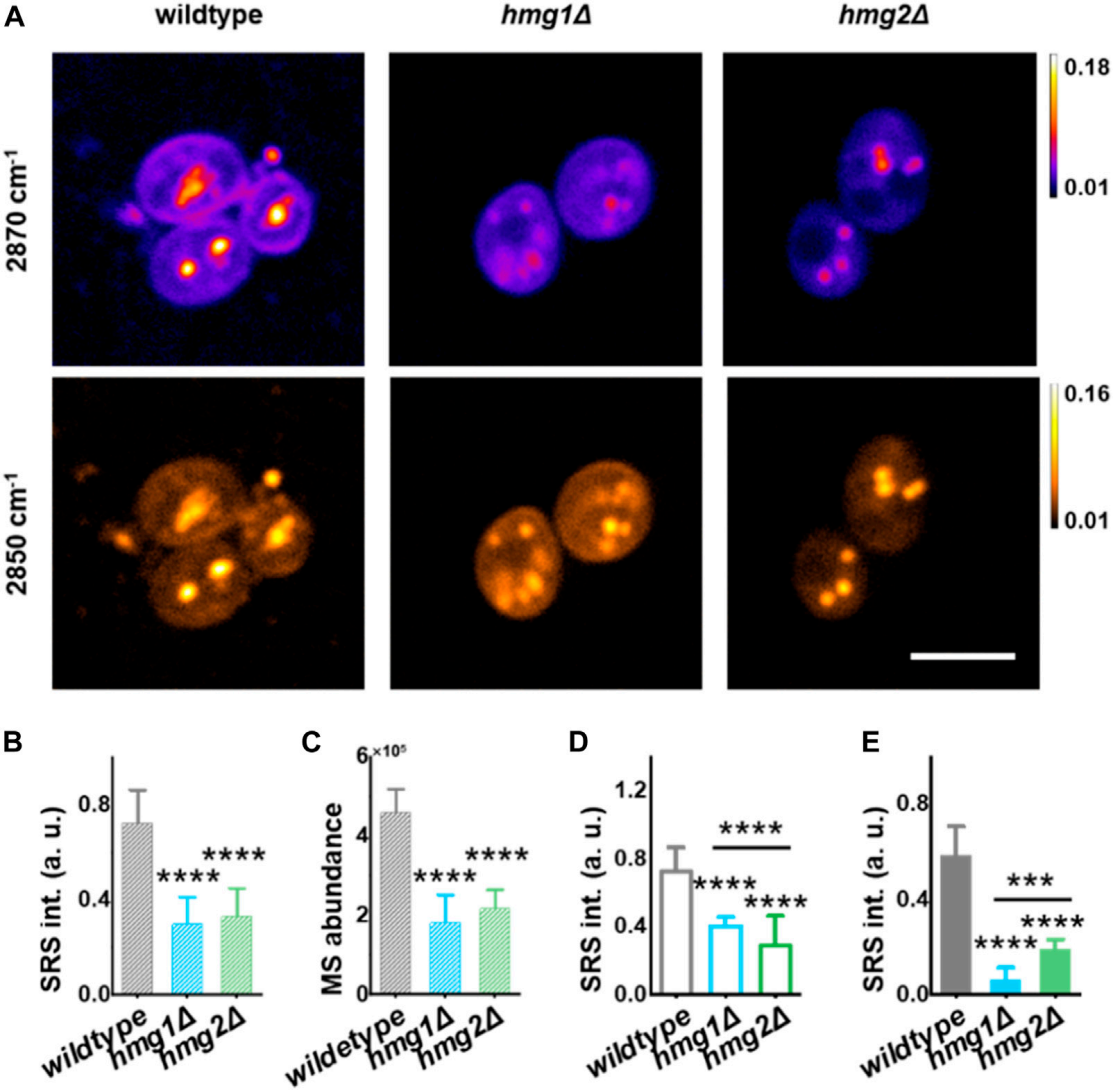
Regulation of ergosterol at the subcellular level by HMGR isozymes revealed by SRS-GEM. **(A)** Representative SRS images at 2,870 cm^−1^ and 2,850 cm^-1^ in *S. cerevisiae* strains. **(B)** Total ergosterol SRS intensity collected from a single *S. cerevisiae* cell obtained by dual-color unmixing analysis. (*n* = 10 for wildtype, *n* = 9 for *hmg1Δ*, and *n* = 14 for *hmg2Δ*). **(C)** Total ergosterol abundance extracted by mass spectrometry. MS, mass spectrometry. **(D)** Ergosterol signal from the plasma membrane extracted by dual-color unmixing analysis. (*n* = 30 for wildtype, *n* = 24 for *hmg1Δ*, *n* = 31 for *hmg2Δ*). **(E)** Ergosterol content in individual LD obtained by dual-color unmixing analysis (wildtype, *n* = 11; *hmg1Δ*, *n* = 16; *hmg2Δ*, *n* = 26). Error bars represent +SD. *, *p* < 0.05; **, *p* < 0.01; ***, *p* < 1 × 10^−3^; ****, *p* < 1 × 10^−4^. Scale bar: 5 μm.

We performed sterol imaging of the genetically modulated strains and cross-validated them by mass spectrometry (MS). Genetic modulation was constructed by knocking out HMGR, i.e., *hmg1Δ* and *hmg2Δ* strains (Supplementary Figures S1A, B), as cells cannot survive with simultaneous knockout (KO) of *HMG1* and *HMG2* (Basson et al., 1986). SRS-GEM images of ergosterol in wildtype showed strong signals in LDs and cellular membranes, while in the KO strains, significantly lower signals were observed (Figure 2A, at 2,870 cm^-1^). Furthermore, quantifying the SRS signal intensity, total ergosterol from *hmg1Δ* and *hmg2Δ* cells reduced significantly with *p* < 1 × 10^−4^ (Figure 2B). Importantly, the mass spectrometry measurement of ergosterol in a population of yeast cells showed an almost similar trend with *p* < 1 × 10^−4^ (Figure 2C). These results also demonstrate that SRS-GEM imaging allows quantitative measurement of ergosterol at the single-cell level.

### *HMG1* and *HMG2* regulate ergosterol distribution differently

We noticed that ergosterol distribution is different between *hmg1Δ* and *hmg2Δ*. To study the subcellular distribution of ergosterol, we analyzed ergosterol in the plasma membrane and LDs in the SRS-GEM images of single yeast cells (Figure 2A). The SRS signal across the plasma membrane is defined by drawing a line from the extracellular, near-membrane position where the signal crosses the threshold (background +3 standard deviations) for the first time, and the highest SRS intensity positioned at the mid-point of the line (Supplementary Figures S2A, B). As expected, the membrane ergosterol level is the highest in the wildtype compared with other groups. Surprisingly, we found a significant difference in membrane ergosterol levels between the *hmg1Δ* and *hmg2Δ* groups (Figure 2D). Furthermore, while the *hmg1Δ* strain also showed reduction compared to the wildtype, the *hmg2Δ* strain showed a higher reduction than the *hmg1Δ* strain (Figure 2D, *p* < 1 × 10^−4^ between *hmg1Δ* and *hmg2Δ*). Interestingly, the ergosterol levels in LDs showed an opposite trend, while the ergosterol content in LDs showed a higher decrease in the *hmg1Δ* strain; a lower decrease was observed in the *hmg2Δ* strain (Figure 2E, *p* < 1 × 10^−3^ between *hmg1Δ* and *hmg2Δ*).

**FIGURE 3:**
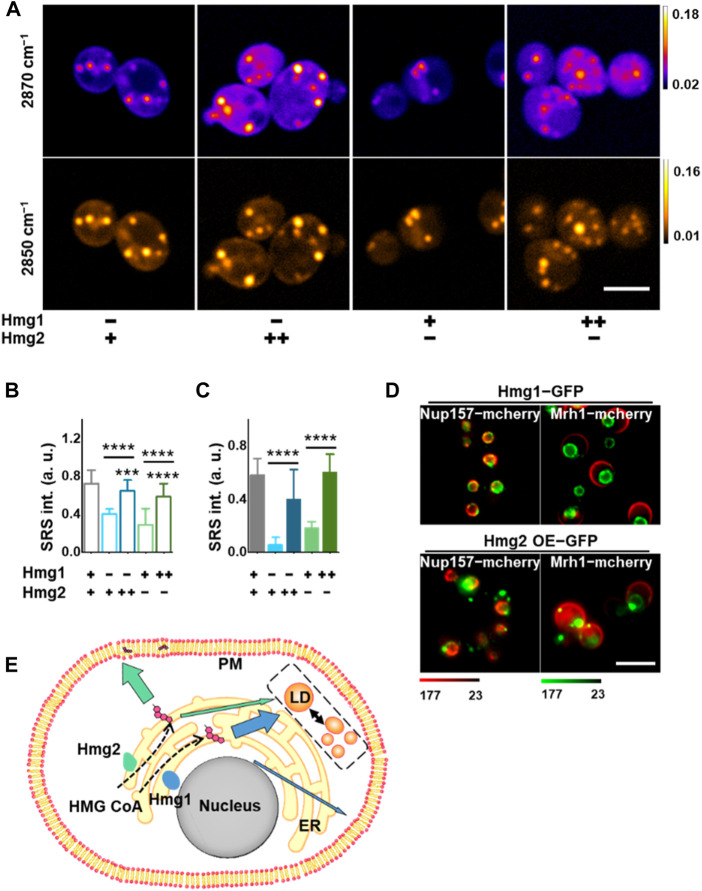
Distinctive subcellular pattern of HMGR isozymes for local ergosterol synthesis. **(A)** Representative SRS images at 2,870 cm^−1^ and 2,850 cm^-1^ in *S. cerevisiae* with different HMGR isozyme expressions. **(B)** Ergosterol signal from the plasma membrane after HMGR knockout or overexpression extracted by dual-color analysis. (*n* = 30, 24, 24, 30, and 30, respectively). Error bars: +SD. **(C)** Ergosterol content in individual LD after HMGR knockout or overexpression extracted by dual-color analysis (*n* = 11, 16, 26, 26, and 26, respectively). Error bars: +SD. **(D)** Confocal fluorescent images of yeast cells for HMGR isozyme distribution. Green: HMGR. Red: Nup157 for the nuclear membrane and Mrh1 for the plasma membrane. **(E)** Proposed regulatory mechanism for lipid and ergosterol metabolism. *, *p* < 0.05; **, *p* < 0.01; ***, *p* < 1 × 10^−3^; ****, *p* < 1 × 10^−4^. Scale bars, 5 μm. “−” stands for KO, “+” stands for the normal level, and “++” stands for OE.

To validate these observations, we overexpressed either Hmg1 or Hmg2 in the KO strains (Supplementary Figures S1A, B) and compared subcellular ergosterol levels using SRS-GEM imaging of single yeast cells (Figure 3A). Both Hmg1 and Hmg2 overexpression (OE) resulted in increased ergosterol levels in the plasma membrane. Importantly, although overexpressing either Hmg1 or Hmg2 rescued the ergosterol level in the plasma membrane, Hmg2 OE in the *hmg1Δ* strain resulted in a membrane ergosterol level similar to wildtype. It suggests that HMG2 mainly contributes to ergosterol distribution in the plasma membrane (Figure 3B; Supplementary Figures S5A, B, *p* < 1 × 10^−4^). At the same time, both Hmg1 OE and Hmg2 OE rescued the ergosterol level in LDs (Figure 3C, *p* < 1 × 10^−4^). It is likely that ergosterol synthesized by *HMG1* is mainly distributed to LDs, given that Hmg1 OE slightly rescued membrane ergosterol levels but resulted in a robust increase in ergosterol in LDs. These results suggest that *HMG1* and *HMG2* regulate ergosterol subcellular distribution differently.

### The subcellular location of HMGRs may regulate ergosterol fate

The aforementioned findings triggered us to investigate the mechanism behind the subcellular regulation of ergosterol by different HMGR isozymes because HMGRs are found to be expressed on the ER membranes (Koning et al., 1996; Gatta et al., 2015). We tracked the expression profiles of *HMG1* and *HMG2* using GFP fusion proteins (method in Supplementary Figure S5C). To validate their locations, the nuclear and plasma membranes were labeled by expressing Nup157-mCherry and Mrh1-mCherry, respectively. From the imaging results (Figure 3D), interestingly, we found a distinctive subcellular expression pattern where *HMG1* is almost exclusively expressed on the peri-nuclear side of ER. In contrast, *HMG2* is primarily expressed on the peri-membrane side.

Based on the aforementioned complementary observations, we suggest an unrecognized regulatory mechanism of sterol synthesis and trafficking, driven by the subcellular enrichment of critical enzymes (Figure 3E). *HMG1* and *HMG2* are expressed differently to promote subcellular enrichment of the rate-limiting step of ergosterol synthesis. Specifically, *HMG1* is mainly expressed on the peri-nuclear ER, which drives ergosterol synthesis and storage in LDs. *HMG2*, on the other hand, is expressed mainly on the peri-membrane ER, which mediates more efficient synthesis and incorporation of ergosterol into the plasma membrane.

### *HMG1* and *HMG2* regulate LD properties differently

In addition to changing the content and distribution of ergosterol, we found that modulating the expression of HMGR also changed the LD properties in yeast. To image lipids, a prominent peak at 2,850 cm^-1^ in the Raman spectrum of olive oil was used (Supplementary Figure S6A), which arises mainly from the CH_2_ symmetric stretch mode. The spectral width of this peak is 22 cm^-1^, which matches the FWHM of the pump and Stokes beams. By SRS-GEM imaging at 2,850 cm^-1^, prominent LD features were observed (Figures 2A, 3A; Supplementary Figure S6B). To quantify different LD properties, SRS signals from LDs were isolated by applying a threshold (Supplementary Figure S6C, Experimental Section).

From quantification, we found that the size of LDs decreased significantly after *HMG2* KO (Supplementary Figure S6D). Although the size of LDs was not rescued, Hmg1 OE in *hmg2Δ* resulted in an increased number of LDs (Supplementary Figure S6E). On the other hand, Hmg2 OE in *hmg1Δ* rescued the LD size (Supplementary Figure S6D). Interestingly, the LDs found in the Hmg2 OE *hmg1Δ* are closer to the plasma membrane than those found in the Hmg1 OE *hmg2Δ* (Supplementary Figure S6F, *p* < 1 × 10^−4^), indicating a local regulation of LD metabolism by HMGR isozymes. Collectively, these results demonstrate distinctive functional roles of *HMG1* and *HMG2*.

### *HMG1* and *HMG2* affect the yeast cell size and survival, respectively

**FIGURE 4:**
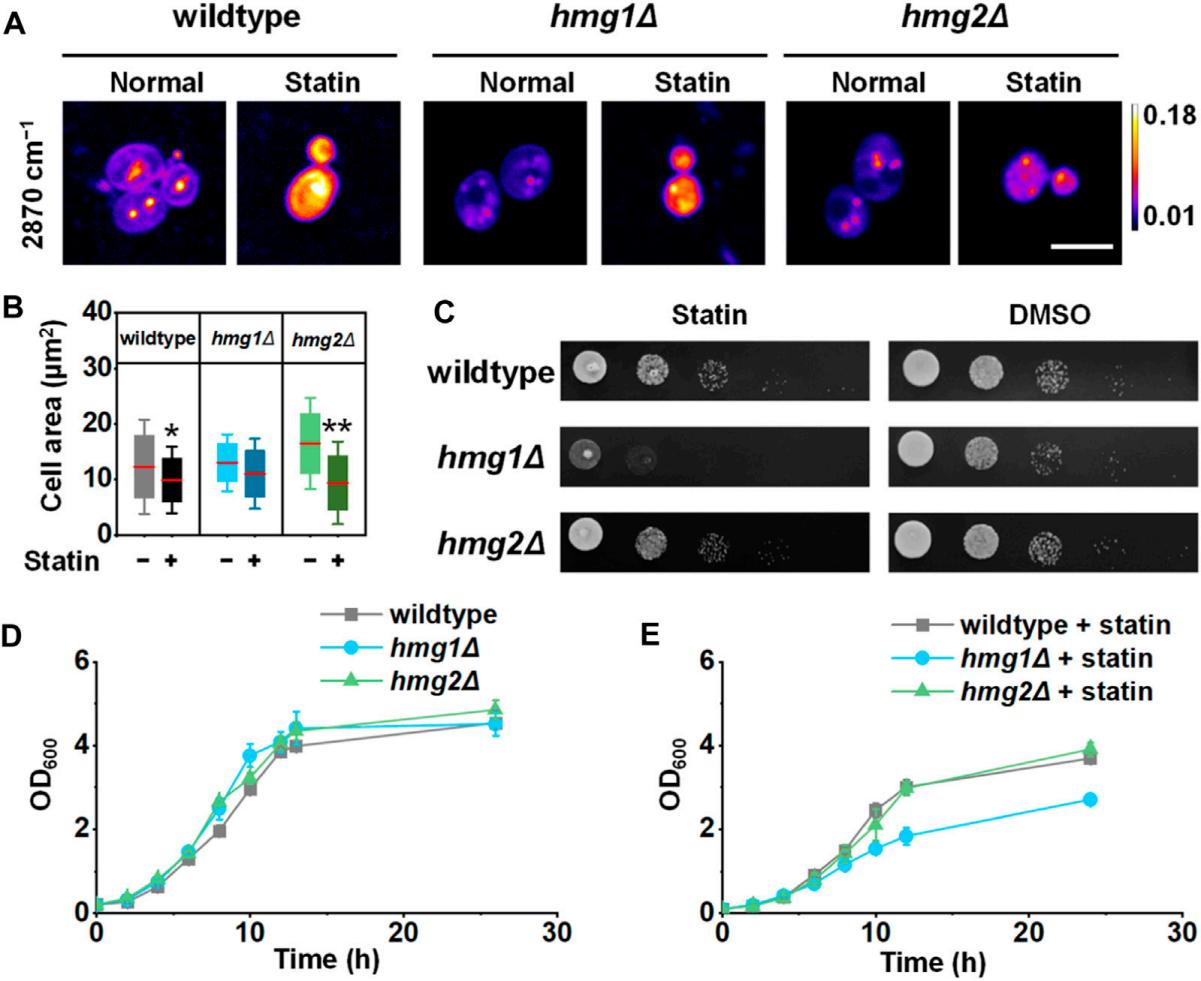
Effects of two HMGR isozymes on the cell size and survival rate. **(A)** Representative SRS images of ergosterol after simvastatin treatment (100 mM, 6 h). **(B)** Quantification of the cell area (*n* = 37, 40, 27, 49, 27, and 40). **(C)** Viability of *S. cerevisiae* after simvastatin treatment compared to the DMSO control. **(D, E)** Growth curves of *S. cerevisiae* with wildtype and simvastatin treatment, respectively, (*n* = 3 for each group). Error bars: ±SD. *, *p* < 0.05; **, *p* < 0.01; ***, *p* < 1 × 10^−3^; ****, *p* < 1 × 10^−4^. Scale bar: 5 μm. “−” stands for untreated and “+” stands for treated.

Ergosterol distribution is essential for cell growth and survival. Therefore, it is likely that spatial regulation of ergosterol synthesis will cause different physiological consequences. Because the complete knockout of both *HMG1* and *HMG2* is lethal, we adopted a potent HMGR inhibitor, simvastatin, to study this process. As a treatment of hypercholesterolemia, simvastatin competitively binds to HMG-coenzyme A with HMGR (Maciejak et al., 2013), reducing the synthesis of cholesterol (or ergosterol in yeast). We found that the cell size and viability reduced significantly after simvastatin treatment in the wildtype strain (Figures 4A–C). Interestingly, the cell size reduction by simvastatin treatment was only observed in the *hmg2Δ* strain (Figure 4B, *p* < 1 × 10^−2^), suggesting *HMG1* may be essential for regulating the cell size. On the other hand, cell viability is suppressed most significantly in the *hmg1Δ* strain by simvastatin (Figures 4C–E), suggesting *HMG2* may be essential for maintaining cell survival.

## Discussion

The spatial regulation of metabolic mechanisms demonstrates the importance of imaging technologies in tracking biomolecules. With the resolution beyond the optical diffraction limit, localization microscopy techniques have broad applications in biology and medicine. However, fluorescence-based imaging relies on fluorophores to selectively tag target molecules, presenting problems when tracking small molecules such as cholesterol or sterol. Furthermore, the amount of dye needed for the super-resolution fluorescence technique increases quadratically with the fold improvement of resolution. Nevertheless, lipid-tracking dyes are usually based on hydrophobicity and, thus, low in selectivity (Yen et al., 2010). Therefore, SRS-GEM provides label-free imaging of mapping small biological molecules and avoids the various problems caused by labeling.

Based on our hypothesized molecular mechanism, *HMG1* primarily drives ergosterol distribution to LDs, an energy storage unit for a cell. In contrast, *HMG2* mainly drives ergosterol distribution to the plasma membrane, where sterol-rich membrane structures, such as lipid rafts, are formed. It is known that lipid rafts are essential for pro-survival signal transduction (George and Wu, 2012). Therefore, the physiological functions of *HMG1* and *HMG2* may be related to the final fate of ergosterol.

We note that protein isoforms often show different metabolic activities, which accompany specific regional distribution at tissue levels. For example, mammalian cells express sterol regulatory element-binding protein (SREBP) isoforms, namely, SREBP-1a, SREBP-1c, and SREBP-2, which show different efficiencies for regulating fatty acid and cholesterol synthesis. Furthermore, SREBP-1c and SREBP-2 are expressed in most tissues, whereas SREBP-1a is mainly expressed in specific tissues and cells, showing a difference in spatial preferences (Shimano et al., 1997). Moreover, lactate dehydrogenase (LDH) has five isoforms, among which LDH-1 and LDH-5 show differential distributions in the brain (Laughton et al., 2000). Our study revealed the regulation of cellular metabolism by different protein isoforms at the subcellular level with a much higher spatial resolution. These results present the importance of precise regulation of ergosterol synthesis and distribution at the subcellular level.

Both ergosterol synthesis and transport are energy-intensive processes (Hu et al., 2017). Therefore, the proposed mechanism here is likely a result of balancing the energy consumption from the synthesis and transport of ergosterol to enhance the efficiency. In previous studies, Koning et al. reported that Hmg2 OE results in the proliferation of the ER membrane (Koning et al., 1996), which will extend toward the plasma membrane. Interestingly, other proteins mediating ergosterol transport, such as ERG28 (Mo et al., 2004), are co-localized in the proliferating ER membranes (Federovitch et al., 2008), suggesting one function of the proliferating ER membrane is to transport ergosterol. More recently, a similar phenomenon has been observed in regulating nucleus–vacuole junctions (NVJs), a multifunctional platform for lipid synthesis. *HMG1* is found to be enriched in the NVJ under acute cellular glucose restriction (Hariri et al., 2018; Rogers et al., 2021). Such enrichment enhanced the efficiency of the mevalonate pathway. Together, these studies further support our hypothesis that subcellular enrichment of HMGR is essential for more efficient sterol synthesis and transport.

Mammalian cells express only one HMGR, so to translate our findings into the mammalian system, we suggest that mammalian HMGR might bind to the peri-nuclear and peri-membrane ER through interactions with different protein complexes or unique post-translational modifications. It indicates that the ER properties at different subcellular locations are essential in achieving subcellular-level cholesterol synthesis regulation. Future studies to explore such a mechanism in the mammalian system and design approaches to control the expression of HMGR at specific subcellular locations will be critical. Overall, these findings present a fundamental mechanism for the precise regulation of subcellular sterol synthesis, potentially inspiring therapeutic approaches for cholesterol-related diseases. Finally, we have demonstrated that SRS-GEM imaging of metabolites at the subcellular level is an enabling technology to provide new insights into metabolic processes.

## Data Availability

The original contributions presented in the study are included in the article/Supplementary Material; further inquiries can be directed to the corresponding authors.

## References

[B1] Argov N. Wachsmann-Hogiu S. Freeman S. L. Huser T. Lebrilla C. B. German J. B. (2008). Size-dependent lipid content in human milk fat globules. J. Agric. Food Chem. 56, 7446–7450. 10.1021/jf801026a 18656925

[B2] Basson M. E. Thorsness M. Rine J. (1986). *Saccharomyces cerevisiae* contains two functional genes encoding 3-hydroxy-3-methylglutaryl-coenzyme A reductase. Proc. Natl. Acad. Sci. U. S. A. 83, 5563–5567. 10.1073/pnas.83.15.5563 3526336 PMC386328

[B3] Berto P. Scotté C. Galland F. Rigneault H. de Aguiar H. B. (2017). Programmable single-pixel-based broadband stimulated Raman scattering. Opt. Lett. 42, 1696–1699. 10.1364/ol.42.001696 28454138

[B4] Burg J. S. Espenshade P. J. (2011). Regulation of HMG-CoA reductase in mammals and yeast. Prog. Lipid Res. 50, 403–410. 10.1016/j.plipres.2011.07.002 21801748 PMC3184313

[B5] Cameron J. S. Moro F. Simmonds H. A. (1993). Gout, uric acid and purine metabolism in paediatric nephrology. Pedia 7, 105–118. 10.1007/bf00861588 8439471

[B6] Casey W. M. Keesler G. A. Parks L. W. (1992). Regulation of partitioned sterol biosynthesis in *Saccharomyces cerevisiae* . J. Bacteriol. 174, 7283–7288. 10.1128/jb.174.22.7283-7288.1992 1429452 PMC207422

[B7] Chen X. Cui S. Yan S. Zhang S. Fan Y. Gong Y. (2021). Hyperspectral stimulated Raman scattering microscopy facilitates differentiation of low-grade and high-grade human prostate cancer. J. Phys. D. 54, 484001. 10.1088/1361-6463/ac2175

[B8] Cheng J. X. Xie X. S. (2015). Vibrational spectroscopic imaging of living systems: An emerging platform for biology and medicine. Science 350, aaa8870. 10.1126/science.aaa8870 26612955

[B9] Choudhary V. Schneiter R. (2012). Pathogen-Related Yeast (PRY) proteins and members of the CAP superfamily are secreted sterol-binding proteins. Proc. Natl. Acad. Sci. U. S. A. 109, 16882–16887. 10.1073/pnas.1209086109 23027975 PMC3479496

[B10] Chung C. Y. Potma E. O. (2013). Biomolecular imaging with coherent nonlinear vibrational microscopy. Annu. Rev. Phys. Chem. 64, 77–99. 10.1146/annurev-physchem-040412-110103 23245525 PMC3965563

[B11] Daum G. Lees N. D. Bard M. Dickson R. (1998). Biochemistry, cell biology and molecular biology of lipids of *Saccharomyces cerevisiae* . Yeast 14, 1471–1510. 10.1002/(sici)1097-0061(199812)14:16<1471:aid-yea353>3.0.co;2-y 9885152

[B12] Dezaki K. Sone H. Yada T. (2008). Ghrelin is a physiological regulator of insulin release in pancreatic islets and glucose homeostasis. Pharmacol 118, 239–249. 10.1016/j.pharmthera.2008.02.008 18433874

[B13] Du J. Wang H. Wei L. (2022). Bringing vibrational imaging to chemical biology with molecular probes. ACS Chem. Biol. 17, 1621–1637. 10.1021/acschembio.2c00200 35772040 PMC10676805

[B14] Federovitch C. M. Jones Y. Z. Tong A. H. Boone C. Prinz W. A. Hampton R. Y. (2008). Genetic and structural analysis of Hmg2p-induced endoplasmic reticulum remodeling in *Saccharomyces cerevisiae* . Mol. Biol. Cell 19, 4506–4520. 10.1091/mbc.e07-11-1188 18667535 PMC2555956

[B15] Freudiger C. W. Min W. Saar B. G. Lu S. Holtom G. R. He C. (2008). Label-free biomedical imaging with high sensitivity by stimulated Raman scattering microscopy. Science 322, 1857–1861. 10.1126/science.1165758 19095943 PMC3576036

[B16] Fu D. Holtom G. Freudiger C. Zhang X. Xie X. S. (2013). Hyperspectral imaging with stimulated Raman scattering by chirped femtosecond lasers. J. Phys. Chem. B 117, 4634–4640. 10.1021/jp308938t 23256635 PMC3637845

[B17] Fu D. Lu F. K. Zhang X. Freudiger C. Pernik D. R. Holtom G. (2012). Quantitative chemical imaging with multiplex stimulated Raman scattering microscopy. J. Am. Chem. Soc. 134, 3623–3626. 10.1021/ja210081h 22316340 PMC3396204

[B18] Gatta A. T. Wong L. H. Sere Y. Y. Calderón-Noreña D. M. Cockcroft S. Menon A. K. (2015). A new family of StART domain proteins at membrane contact sites has a role in ER-PM sterol transport. eLife 4, e07253. 10.7554/elife.07253 26001273 PMC4463742

[B63] George K. S. Wu S. (2012). Lipid raft: A floating island of death or survival. Toxicol. Appl. Pharmacol. 259, 311–319. 10.1016/j.taap.2012.01.007 22289360 PMC3299927

[B19] Gillies R. J. Robey I. Gatenby R. A. (2008). Causes and consequences of increased glucose metabolism of cancers. J. Nucl. Med. 49 (2), 24S–42S. 10.2967/jnumed.107.047258 18523064

[B20] Grundy S. M. Stone N. J. Bailey A. L. Beam C. Birtcher K. K. Blumenthal R. S. (2019). 2018 AHA/ACC/AACVPR/AAPA/ABC/ACPM/ADA/AGS/APhA/ASPC/NLA/PCNA guideline on the management of blood cholesterol. J. Am. Coll. Cardiol. 73, e285–e350. 10.1016/j.jacc.2018.11.003 30423393

[B21] Hariri H. Rogers S. Ugrankar R. Liu Y. L. Feathers J. R. Henne W. M. (2018). Lipid droplet biogenesis is spatially coordinated at ER-vacuole contacts under nutritional stress. EMBO Rep. 19, 57–72. 10.15252/embr.201744815 29146766 PMC5757283

[B22] Hotamisligil G. S. (2006). Inflammation and metabolic disorders. Nature 444, 860–867. 10.1038/nature05485 17167474

[B23] Hu Z. He B. Ma L. Sun Y. Niu Y. Zeng B. (2017). Recent advances in ergosterol biosynthesis and regulation mechanisms in *Saccharomyces cerevisiae* . Indian J. Microbiol. 57, 270–277. 10.1007/s12088-017-0657-1 28904410 PMC5574775

[B24] Huang K. C. Li J. Zhang C. Tan Y. Cheng J. X. (2020). Multiplex stimulated Raman scattering imaging cytometry reveals lipid-rich protrusions in cancer cells under stress condition. iScience 23, 100953. 10.1016/j.isci.2020.100953 32179477 PMC7078382

[B25] Jung Y. Y. Ko J. H. Um J. Y. Chinnathambi A. Alharbi S. A. Sethi G. (2021). LDL cholesterol promotes the proliferation of prostate and pancreatic cancer cells by activating the STAT3 pathway. J. Cell. Physiol. 236, 5253–5264. 10.1002/jcp.30229 33368314

[B26] Karanja C. W. Hong W. Younis W. Eldesouky H. E. Seleem M. N. Cheng J. X. (2017). Stimulated Raman imaging reveals aberrant lipogenesis as a metabolic marker for azole-resistant Candida albicans. Anal. Chem. 89, 9822–9829. 10.1021/acs.analchem.7b01798 28813144

[B27] Kaul D. (2003). Cholesterol-receptor-mediated genomics in health and disease. Trends. Mol. Med. 9, 442–449. 10.1016/j.molmed.2003.08.010 14557057

[B28] Kishida T. Kostetskii I. Zhang Z. Martinez F. Liu P. Walkley S. U. (2004). Targeted mutation of the MLN64 START domain causes only modest alterations in cellular sterol metabolism. J. Biol. Chem. 279, 19276–19285. 10.1074/jbc.m400717200 14963026

[B29] Koning A. J. Roberts C. J. Wright R. L. (1996). Different subcellular localization of *Saccharomyces cerevisiae* HMG-CoA reductase isozymes at elevated levels corresponds to distinct endoplasmic reticulum membrane proliferations. Mol. Biol. Cell 7, 769–789. 10.1091/mbc.7.5.769 8744950 PMC275929

[B30] Laughton J. D. Charnay Y. Belloir B. Pellerin L. Magistretti P. J. Bouras C. (2000). Differential messenger RNA distribution of lactate dehydrogenase LDH-1 and LDH-5 isoforms in the rat brain. Neuroscience 96, 619–625. 10.1016/s0306-4522(99)00580-1 10717443

[B31] Lee H. J. Zhang W. Zhang D. Yang Y. Liu B. Barker E. L. (2015). Assessing cholesterol storage in live cells and *C. elegans* by stimulated Raman scattering imaging of phenyl-diyne cholesterol. Sci. Rep. 5, 7930. 10.1038/srep07930 25608867 PMC4302291

[B32] Liao C. S. Wang P. Wang P. Li J. Lee H. J. Eakins G. (2015). Spectrometer-free vibrational imaging by retrieving stimulated Raman signal from highly scattered photons. Sci. Adv. 1, e1500738. 10.1126/sciadv.1500738 26601311 PMC4646825

[B33] Lin H. Lee H. J. Tague N. Lugagne J. B. Zong C. Deng F. (2021). Microsecond fingerprint stimulated Raman spectroscopic imaging by ultrafast tuning and spatial-spectral learning. Nat. Commun. 12, 3052. 10.1038/s41467-021-23202-z 34031374 PMC8144602

[B34] Lin H. Liao C. S. Wang P. Kong N. Cheng J. X. (2018). Spectroscopic stimulated Raman scattering imaging of highly dynamic specimens through matrix completion. Light Sci. Appl. 7, 17179. 10.1038/lsa.2017.179 30839525 PMC6060072

[B35] Maciejak A. Leszczynska A. Warchol I. Gora M. Kaminska J. Plochocka D. (2013). The effects of statins on the mevalonic acid pathway in recombinant yeast strains expressing human HMG-CoA reductase. BMC Biotechnol. 13, 68. 10.1186/1472-6750-13-68 24128347 PMC3765880

[B36] Min W. Freudiger C. W. Lu S. Xie X. S. (2011). Coherent nonlinear optical imaging: Beyond fluorescence microscopy. Annu. Rev. Phys. Chem. 62, 507–530. 10.1146/annurev.physchem.012809.103512 21453061 PMC3427791

[B37] Mo C. Valachovic M. Bard M. (2004). The ERG28-encoded protein, Erg28p, interacts with both the sterol C-4 demethylation enzyme complex as well as the late biosynthetic protein, the C-24 sterol methyltransferase (Erg6p). Biochim. Biophys. Acta Biomembr. 1686, 30–36. 10.1016/j.bbalip.2004.08.001 15522820

[B38] Mok H. Y. von Bergmann K. Grundy S. M. (1979). Effects of continuous and intermittent feeding on biliary lipid outputs in man: Application for measurements of intestinal absorption of cholesterol and bile acids. J. Lipid Res. 20, 389–398. 10.1016/s0022-2275(20)40622-4 109556

[B39] Okotrub K. A. Shamaeva D. V. Surovtsev N. V. (2022). Raman spectra of deuterated hydrocarbons for labeling applications. J. Raman Spectrosc 53, 297–309. 10.1002/jrs.6279

[B40] Peckett A. J. Wright D. C. Riddell M. C. (2011). The effects of glucocorticoids on adipose tissue lipid metabolism. Metab 60, 1500–1510. 10.1016/j.metabol.2011.06.012 21864867

[B41] Porter J. A. Young K. E. Beachy P. A. (1996). Cholesterol modification of hedgehog signaling proteins in animal development. Science 274, 255–259. 10.1126/science.274.5285.255 8824192

[B42] Ramkumar S. Raghunath A. Raghunath S. (2016). Statin therapy: Review of safety and potential side effects. Acta Cardiol. Sin. 32, 631–639. 10.6515/acs20160611a 27899849 PMC5126440

[B43] Riscal R. Skuli N. Simon M. C. (2019). Even cancer cells watch their cholesterol. Mol. Cell. 76, 220–231. 10.1016/j.molcel.2019.09.008 31586545 PMC7225778

[B44] Rogers S. Hariri H. Wood N. E. Speer N. O. Henne W. M. (2021). Glucose restriction drives spatial reorganization of mevalonate metabolism. eLife 10, e62591. 10.7554/elife.62591 33825684 PMC8057812

[B45] Sakamoto K. Kimura J. (2013). Mechanism of statin-induced rhabdomyolysis. J. Pharmacol. Sci. 123, 289–294. 10.1254/jphs.13r06cp 24257439

[B46] Shimano H. Shimomura I. Hammer R. E. Herz J. Goldstein J. L. Brown M. S. (1997). Elevated levels of SREBP-2 and cholesterol synthesis in livers of mice homozygous for a targeted disruption of the SREBP-1 gene. J. Clin. Invest. 100, 2115–2124. 10.1172/jci119746 9329978 PMC508404

[B47] Söderholm S. Roos Y. H. Meinander N. Hotokka M. (1999). Raman spectra of fructose and glucose in the amorphous and crystalline states. J. Raman Spectrosc 30, 1009–1018. 10.1002/(sici)1097-4555(199911)30:11<1009:aid-jrs436>3.0.co;2-#

[B48] Van Dijken J. P. Bauer J. Brambilla L. Duboc P. Francois J. M. Gancedo C. (2000). An interlaboratory comparison of physiological and genetic properties of four *Saccharomyces cerevisiae* strains. Enzyme Microb. Technol. 26, 706–714. 10.1016/s0141-0229(00)00162-9 10862876

[B49] Vance J. E. Hayashi H. Karten B. (2005). Cholesterol homeostasis in neurons and glial cells. Semin. Cell Dev. Biol. 16, 193–212. 10.1016/j.semcdb.2005.01.005 15797830

[B50] Wei L. Hu F. Chen Z. Shen Y. Zhang L. Min W. (2016). Live-cell bioorthogonal chemical imaging: Stimulated Raman scattering microscopy of vibrational probes. Accounts Chem. Res. 49, 1494–1502. 10.1021/acs.accounts.6b00210 PMC5704954 27486796

[B51] Wei L. Hu F. Shen Y. Chen Z. Yu Y. Lin C.-C. (2014). Live-cell imaging of alkyne-tagged small biomolecules by stimulated Raman scattering. Nat. methods 11, 410–412. 10.1038/nmeth.2878 24584195 PMC4040164

[B52] Wei L. Shen Y. Xu F. Hu F. Harrington J. K. Targoff K. L. (2015). Imaging complex protein metabolism in live organisms by stimulated Raman scattering microscopy with isotope labeling. ACS Chem. Biol. 10, 901–908. 10.1021/cb500787b 25560305 PMC4610303

[B53] Yamauchi Y. Rogers M. A. (2018). Sterol metabolism and transport in atherosclerosis and cancer. Front. Endocrinol. 9, 509. 10.3389/fendo.2018.00509 PMC6157400 30283400

[B54] Ye C. Bandara W. M. Greenberg M. L. (2013). Regulation of inositol metabolism is fine-tuned by inositol pyrophosphates in *Saccharomyces cerevisiae* . J. Biol. Chem. 288, 24898–24908. 10.1074/jbc.m113.493353 23824185 PMC3750184

[B55] Yen K. Le T. T. Bansal A. Narasimhan S. D. Cheng J. X. Tissenbaum H. A. (2010). A comparative study of fat storage quantitation in nematode *Caenorhabditis elegans* using label and label-free methods. PLoS One 5, e12810. 10.1371/journal.pone.0012810 20862331 PMC2940797

[B56] Yue S. Li J. Lee S. Y. Lee H. J. Shao T. Song B. (2014). Cholesteryl ester accumulation induced by PTEN loss and PI3K/AKT activation underlies human prostate cancer aggressiveness. Cell Met. 19, 393–406. 10.1016/j.cmet.2014.01.019 PMC3969850 24606897

[B57] Yutuc E. Angelini R. Baumert M. Mast N. Pikuleva I. Newton J. (2020). Localization of sterols and oxysterols in mouse brain reveals distinct spatial cholesterol metabolism. Proc. Natl. Acad. Sci. U. S. A. 117, 5749–5760. 10.1073/pnas.1917421117 32132201 PMC7084107

[B58] Zhang D. Slipchenko M. N. Cheng J. X. (2011). Highly sensitive vibrational imaging by femtosecond pulse stimulated Raman loss. J. Phys. Chem. Lett. 2, 1248–1253. 10.1021/jz200516n 21731798 PMC3124560

[B59] Zhang D. Slipchenko M. N. Leaird D. E. Weiner A. M. Cheng J.-X. (2013). Spectrally modulated stimulated Raman scattering imaging with an angle-to-wavelength pulse shaper. Opt. express 21, 13864–13874. 10.1364/oe.21.013864 23736639 PMC3686469

[B60] Zhang D. Wang P. Slipchenko M. N. Cheng J. X. (2014). Fast vibrational imaging of single cells and tissues by stimulated Raman scattering microscopy. Acc. Chem. Res. 47, 2282–2290. 10.1021/ar400331q 24871269 PMC4139189

[B61] Zhou Q. Liao J. K. (2009). Statins and cardiovascular diseases: From cholesterol lowering to pleiotropy. Curr. Pharm. Des. 15, 467–478. 10.2174/138161209787315684 19199975 PMC2896785

