# Supplementary material for: Spatial sterol metabolism unveiled by stimulated Raman imaging
Source: Front Chem. 2023 Mar 29;11:1166313. doi: 10.3389/fchem.2023.1166313 (PMC10090450; doi:10.3389/fchem.2023.1166313)
Supplement: Supplementary file 1 [file DataSheet1.docx]

Supplementary Material

Spatial sterol metabolism unveiled by stimulated Raman imaging

Yongqing Zhang^†^, Yihui Zhou^†^, Wen Fang^†^, Hanlin Zhu, Cunqi Ye^*^, Delong Zhang^*^, and Hyeon Jeong Lee^*^

*** Correspondence:** yecunqi@zju.edu.cn, hjlee@zju.edu.cn, [dlzhang@zju.edu.cn](mailto:dlzhang@zju.edu.cn)

**Supplementary Figures**

Figure S1: HMGR relative abundance of different types obtained by qPCR.

Figure S2: Measuring ergosterol in the plasma membrane through SRS images at 2870 cm^-1^.

Figure S3. The spatial resolution of the SRS-GEM platform.

Figure S4: Peak fitting results of ergosterol spectrum and MATLAB simulation of different spectral resolutions of excitation beams.

Figure S5: Measuring ergosterol in the plasma membrane through SRS images at 2870 cm^-1^ after HMGR OE.

Figure S6: HMGR isozymes regulate LDs properties.

**Figure S1. HMGR relative abundance of different types obtained by qPCR.** (a) Hmg1 relative abundance of different types obtained by qPCR. n = 4 for each group. (b) Hmg2 relative abundance of different types obtained by qPCR. n = 4 for each group. Error bars represent + SD. *, p < 0.05. **, p < 0.01. ***, p < 1 × 10^−3^. ****, p < 1 × 10^−4^. “−” stands for KO, “+” stands for normal level, “++” stands for OE.

**Figure S2. Measuring ergosterol in the plasma membrane through SRS images at 2870 cm^-1^.** (a) Representative SRS images of ergosterol (2870 cm^-1^) in *S. cerevisiae* yeast with HMGR KO. Green lines indicate six points selected equidistantly throughout the whole membrane for analysis. (b) Ergosterol signal across the plasma membrane at 2870 cm^-1^ (as indicated by red lines in (a)). Shaded areas represent the membrane portion. “SD” stands for standard deviation. Scale bar, 5 μm.

**Figure S3. The spatial resolution of the SRS-GEM platform.** (a) SRS image of 200-nm PMMA beads at 2957 cm^-1^. (b) High spatial resolution resolved down to 456 nm as shown from the FWHM of beads in (a). Scale bar, 2 μm.

**Figure S4. Peak fitting results of ergosterol spectrum and MATLAB simulation of different spectral resolutions of excitation beams.** (a) The Raman peak fitting of ergosterol spectrum. (b) The Raman peaks and the Gaussian function for simulating the spectral resolution of 10 cm^-1^ at 2870 cm^-1^. (c) the result spectra gotten of 10 cm^-1^ spectral resolution by MATLAB simulation. (d) The SBR of excitation beams with different spectral resolutions. The blue arrows indicated the ratios when spectral resolutions were 10 cm^-1^ and 40 cm^-1^. SBR: signal-to-background ratio. SR: spectral resolution.

**Figure S5.** **Measuring ergosterol in the plasma membrane through SRS images at 2870 cm^-1^ after HMGR OE.** (a) Representative SRS images of ergosterol (2870 cm^-1^) in *S. cerevisiae* with HMGR OE. (b) Ergosterol signal across the plasma membrane at 2870 cm^-1^ (as indicated by red lines in (a)). Shaded areas represent the membrane portion. “SD” stands for standard deviation. (c) Design schematic diagram of Hmg1-GFP and Hmg2-GFP labeled yeast strains. Scale bar, 5 μm. “−” stands for KO, “+” stands for normal level, “++” stands for OE.

**Figure S6.** **HMGR isozymes regulate LDs properties.** (a) Raman spectrum of lipid. The Gray area highlights the characteristic peak selected for visualizing lipid, with the width indicating the spectral resolution of our SRS system, 10 cm^-1^. Acquisition time: 30s. (b) Representative SRS images of lipid (2850 cm^−1^) in *S. cerevisiae* with HMGR KO. (c) Representative LDs SRS images in *S. cerevisiae* after threshold processing with different HMGR isozyme expressions. Orange lines represent cell outlines. (d) LD size of different groups in *S. cerevisiae*. (n = 19, 25, 43, 43, 26) (e) Statistical analysis of LD numbers per cell in *S. cerevisiae*. (n = 5, 4, 7, 5, 5). (f) Statistical analysis of the distance between LDs and plasma membrane. (n = 69, 43) Error bars represent + SD. *, p < 0.05. **, p < 0.01. ***, p < 1 × 10^−3^. ****, p < 1 × 10^−4^. Scale bars, 5 μm. “−” stands for KO, “+” stands for normal level, “++” stands for OE.
